# A gating mechanism for Pi release governs the mRNA unwinding by eIF4AI during translation initiation

**DOI:** 10.1093/nar/gkv1033

**Published:** 2015-10-12

**Authors:** Junyan Lu, Chenxiao Jiang, Xiaojing Li, Lizhi Jiang, Zengxia Li, Tilman Schneider-Poetsch, Jianwei Liu, Kunqian Yu, Jun O. Liu, Hualiang Jiang, Cheng Luo, Yongjun Dang

**Affiliations:** 1 Key Laboratory of Metabolism and Molecular Medicine, the Ministry of Education, Department of Biochemistry and Molecular Biology, School of Basic Medical Sciences, Fudan University, Shanghai 200032, China; 2 Drug Discovery and Design Center, State Key Laboratory of Drug Research, Shanghai Institute of Materia Medica, Chinese Academy of Sciences, Shanghai 201203, China; 3 Chemical Genetics Laboratory, RIKEN, Wako, Saitama, 351-0198, Japan; 4 Department of Chemistry, Shanghai Key Lab of Chemical Biology for Protein Research & Institutes of Biomedical Sciences, Fudan University, Shanghai 200433, China; 5 Department of Pharmacology & Molecular Sciences and Department of Oncology, Johns Hopkins University School of Medicine, Baltimore, MD 21205, USA

## Abstract

Eukaryotic translation initiation factor eIF4AI, the founding member of DEAD-box helicases, undergoes ATP hydrolysis-coupled conformational changes to unwind mRNA secondary structures during translation initiation. However, the mechanism of its coupled enzymatic activities remains unclear. Here we report that a gating mechanism for Pi release controlled by the inter-domain linker of eIF4AI regulates the coupling between ATP hydrolysis and RNA unwinding. Molecular dynamic simulations and experimental results revealed that, through forming a hydrophobic core with the conserved SAT motif of the N-terminal domain and I357 from the C-terminal domain, the linker gated the release of Pi from the hydrolysis site, which avoided futile hydrolysis cycles of eIF4AI. Further mutagenesis studies suggested this linker also plays an auto-inhibitory role in the enzymatic activity of eIF4AI, which may be essential for its function during translation initiation. Overall, our results reveal a novel regulatory mechanism that controls eIF4AI-mediated mRNA unwinding and can guide further mechanistic studies on other DEAD-box helicases.

## INTRODUCTION

The DEAD-box family of proteins (DBPs) catalyzes the local conformational changes of RNA in an ATP-dependent manner and plays essential roles in all facets of RNA metabolism, including transcription, RNA splicing and editing, RNA transport, ribosome biogenesis, protein translation and RNA degradation (1). The eukaryotic translation initiation factor 4A1 (eIF4AI) is the prototypical DEAD-box helicase, which possess only the highly conserved helicase core (2). Upon incorporation of eIF4A into a complex with eIF4E and eIF4G, the resultant eIF4F complex directly binds to the 5′-cap of mRNA through eIF4E, recruits the small 40S ribosomal subunit via eIF4G and unwinds secondary structures at the 5′ UTR of the mRNA, enabling translation initiation (3). Several natural products with anticancer activity, including pateamine A, hippuristanol and silvestrol have been identified as specific inhibitors of eIF4A, suggesting that eIF4A may serve as a novel target for anticancer drugs (4–6). Recently, it was reported that several essential genes for tumorigenesis, such as c-Myc, have G-quadruplexes in the 5′ UTR of their mRNAs, rendering them highly dependent on the enzymatic activity of eIF4AI for translation initiation (7), further supporting the notion that eIF4AI can be targeted for cancer therapy.

The importance of eIF4AI and other DBPs in normal biological and pathological processes has drawn attentions toward their catalytic mechanisms. Nearly three decades of biochemical, biophysical and structural studies have led to the recognition of some common principles in DBPs (8). The helicase core of all DBPs consists of two rigid RecA-like domains connected by a flexible linker. Opening and closing of the two domains, which is driven by the ATP binding and hydrolysis cycle, is thought to be critical for helicase activities of DBPs (9). DBPs are believed to unwind RNA through local strand separation instead of translocation on RNA, which is a key feature that distinguishes DEAD-box helicases from DNA helicases (10). Nevertheless, the mechanism by which ATP hydrolysis is coupled to helicase activity has only begun to be unraveled in recent years. Allosteric networks formed by several conserved DBP motifs within the helicase core are suggested to meditate the communication between ATP- and RNA-binding sites (9,11). Among these motifs, motif III (SAT) is thought to play a central role in coupling hydrolysis and duplex unwinding, as the mutation of SAT to AAA in eIF4AI decouples ATPase and helicase activity (12). However, the precise coupling and decoupling mechanisms still remain largely unknown. The inter-domain linker, which is less conserved in DBPs, has also been shown to regulate eIF4AI's enzymatic activity, but its role in ATPase and helicase coupling has not been further explored (13). It has been proposed that the closed conformation triggered by nucleotide binding rather than hydrolysis might be critical for duplex unwinding (14). Recent kinetic studies, however, led to the proposition that the post-hydrolysis ADP-Pi-bound state might be the actual working state (15–17). These contradictory mechanistic models further compounded the complexity of DBPs unwinding mechanisms.

Previous studies of the DBPs have relied on mutagenesis, X-ray crystallography and fluorescence resonance energy transfer (FRET) assays (12,18,19). However, these approaches are not sufficient to connect changes in activity and structure, as crystal structures only capture static snapshots during catalytic cycle while FRET experiments, although ideal for studying dynamic conformational change, lacks resolution at the atomic level. To fill the void, computational approaches, such as molecular dynamic (MD) simulations, provide complementary tools for understanding functional dynamics of proteins (20,21). In this study, we investigated the catalytic mechanism of eIF4AI through a combination of MD simulations and enzymatic assays. We found that the hydrophobic core formed by the conserved SAT motif and the inter-domain linker regulates the phosphate release in the post-hydrolysis ADP-Pi binding state as well as the coupling between ATPase and helicase activity of eIF4AI. Further characterizations of the inter-domain linker revealed a diverse role of this non-conserved linker in fine-tuning the helicase unwinding and translation initiation activities of eIF4AI. The new mechanistic insights have important implications in understanding the mechanism of coupling of ATP hydrolysis and RNA unwinding activity of other members of the DBP family.

## MATERIALS AND METHODS

### Homology modeling and canonical molecular dynamic (cMD) simulation

The structure of eIF4AI in the closed conformational state was built using Build Homology Models protocol in Discovery Studio 3.0 (22) and the crystal structure of eIF4AIII bound to AMPPNP and polyU (PDBID: 2HYI) (23) was used as the template. Although this crystal structure represents the inhibited form of eIF4AIII within the exon junction complex (EJC), a previous study indicates eIF4AIII within EJC is hydrolysis-competent but the conformational changes after ATP hydrolysis is blocked by other EJC components (24). By comparing the structure of eIF4AIII within EJC and other DDX structures, including Mss116p (PDBID: 3I5X), Vasa (PDBID: 2DB3) and Dbp5 (PDBID: 3FHT), in the closed conformation, we found the helicase core structures of these proteins were almost the same, and therefore any crystal structure of DDX protein in the closed state could be used to model the closed state eIF4AI theoretically since eIF4AI only possesses the conserved helicase core. The crystal structure of eIF4AIII in the EJC complex was chosen as the template because eIF4AI and eIF4AIII share the highest sequence similarity and eIF4AIII also demonstrates similar *in vitro* enzymatic activity as eIF4AI (25). The AMPPNP in the crystal structure was changed to ATP or ADP by manually modifying or deleting corresponding atoms. To model the ADP+Pi binding state, the γ-phosphate on ATP was replaced by Pi (H_2_PO_4_^−^), followed by a brief energy minimization. The protonation states of the titratable residues under physical conditions were predicted by H++ 3.0 (26). Each complex model was solvated by a cubic water box with its boundary extended 10 Å away from the solute on all side. K^+^ and Cl^−^ were added to neutralize the simulation system and to make the salt concentration close to 0.1 mM. AMBER10 force field (AMBER99SB+ parmbsc0) (27,28) was used for the protein and RNA part. Force field parameters for ATP, ADP and Mg^2+^ were obtained from the AMBER parameter database (www.pharmacy.manchester.ac.uk/bryce/amber). The force field parameters for Pi were taken from previous studies (29). Water molecules were described with the TIP3P model (30). All MD simulations were performed using the Gromacs 4.5.5 package with standard periodic boundary conditions (31). After energy minimization and equilibration, 1 μs production cMD run with a time step of 2 fs was performed for each complex model.

### Locally enhanced sampling (LES) simulation

LES simulations were performed using the Amber 11 package (32,33). Structures taken from cMD trajectories of the eIF4AI−ADP+Pi model and eIF4AI^SAT/AAA^−ADP+Pi model were used as initial structures for LES simulations. The setup of the system was the same as in the cMD simulation. To avoid distortion of the Pi geometry due to high temperature, all Pi force constants were doubled. Before LES simulation, 10 ns cMD equilibrations using Amber 11 was performed for each model. Thirty Pi replicates were used in all LES simulations, similar to previous studies on Pi release from myosin (21). Different to the myosin study, the Pi replicates were only heated up to 700 K because higher temperatures were found to significantly distort the Pi geometry. As our LES simulation time is relatively long (1 ns in our study and 100 ps in the study of Pi release from myosin), a significant number of Pi release events could be observed under 700 K. Due to the spontaneous nature of Pi release event, a total of 30 explicit-water LES simulations at each temperature were performed to obtain statistically meaningful results. Pi positions were analyzed from the last frame of the 1 ns simulation and Pi release was based on a distance criterion using a cutoff of 15 Å between the phosphorus atom and its initial position. The probability of Pi release at each temperature was calculated as the number of Pi replicas released from the protein divided by 900 (30 replicas multiplied by 30 runs).

### Reagents

Antibodies were purchased from various commercial sources: eIF4AI, Santa Cruz; eIF4G, BD Biosciences; eIF4E, Santa Cruz; HRP-conjugated secondary antibodies, Jackson ImmunoResearch.

### Plasmid constructs

Human 6×His-eIF4AI was subcloned into the pET28a vector for recombinant protein purification or pcDNA3.1/HisA vector for overexpression in mammalian cell. eIF4AI mutagenesis was performed by two steps of PCR with primers as listed in Supplementary Table S1 or Quick Change Site-directed mutagenesis to construct the pcDNA3.1/HisA-eIF4AI mutants. All mutants were confirmed by sequencing.

### Recombinant proteins

All recombinant proteins were produced by expression in *E.coli* BL21(DE3) cells (Novagen) by induction with 1 mM IPTG. After growth and harvesting, cells were lysed in lysis buffer by sonication. For His-tagged proteins, purification was performed using Ni-NTA resin (QIAGEN) at 4°C. After purification, all proteins were stored in buffer containing 20 mM Tris (pH 7.4), 100 mM KCl, 0.1 mM EDTA and 10% glycerol (eIF4AI storage buffer). Protein concentrations were determined by Pierce®BCA Protein Assay Kit (Thermo) and protein purity was monitored by SDS-PAGE and Coomassie blue staining.

### ATPase assay

The assay procedure was adopted from previous reports with modifications indicated below (34). Colorimetric determination of eIF4AI ATPase activity was performed as follows: 0.4 μM 6×His-eIF4AI or corresponding mutants were incubated with reaction buffer, which contains 100 mM Tris-HCl (pH 7.5), 6 mM MgCl_2_, 20 mM KCl, 0.01% Triton-X100 and saturated polyU (250 μg/ml). An aliquot (15 μl) of this mixture was added into each well of a 96-well plate, then 10 μl ATP with different concentration was added into each well to start the reactions. After the incubation of the reaction mixture for 30 min at 37°C, an aliquot of 80 μl of malachite green reagent was added to each well and 10 μl 34% sodium citrate was immediately used to stop the hydrolysis of ATP. Samples were mixed thoroughly and incubated at 37 °C for another 15 min. Absorbance of each well at OD620 was then measured by a FlexStation3 (Molecular Devices). Calculations of K_m_ and K_cat_ were determined by curve fitting (based on Michealis–Menten model) using GraphPad Prism 5 software.

### Helicase assay

Fluorescent reporter and loading RNA oligonucleotides were chemically synthesized, modified and HPLC-purified by Integrated DNA Technologies (IDT). The reporter strand was modified with cyanine 3 (Cy3) on its 5′-end, and the loading strand was modified with a spectrally paired black hole quencher (BHQ) on its 3′-end. The sequences of Cy3-labeled RNA, BHQ-labeled RNA, and ‘competitor’ DNA are shown, respectively, with underlined base pairs signifying a duplex region: reporter strand (5′-Cy3-GCUUUACGGUGC-3′); quenching strand (5′-GAACAACAACAACAACCAUGGCACCGUAAAGC-BHQ-3′); and DNA capture strand (5′-GCACCGTAAAGC-3′). Unwinding reactions were performed in a 96 wells RNase free white plate (Thermal) by using an EnVision® Multilabel Reader (PerkinElmer). Cy3-labeled RNA was annealed to BHQ-labeled RNA oligos, forming a duplex region with a 5′-overhang that possesses low fluorescence. Duplex substrate was incubated with different mutations of recombinant eIF4AI (1 μM), and the unwinding reaction was initiated by the addition of 2 mM ATP-Mg^2+^ at 37°C. The change in fluorescence was calibrated to the fraction of duplex unwound over time (35).

### *In vitro* translation assay

Flexi® Rabbit Reticulocyte Lysate System was purchased from Promega. Reactions contained 2 μM 6×His-eIF4AI or mutants in a 10-μl system at 30°C for 60 min. Luciferase RNA in the kit was used as *In vitro* translation reporter and luciferase activity was measured with Envision (PerkinElmer).

### Statistical analysis

Biochemical experiments were repeated three times at least. Values are reported as the Means ± SEM (standard error of mean) and analyzed using two-tailed Student's t-test.

## RESULTS

### ATP-to-ADP transition leads to conformational instability of eIF4AI in the closed state

**Figure 1.**
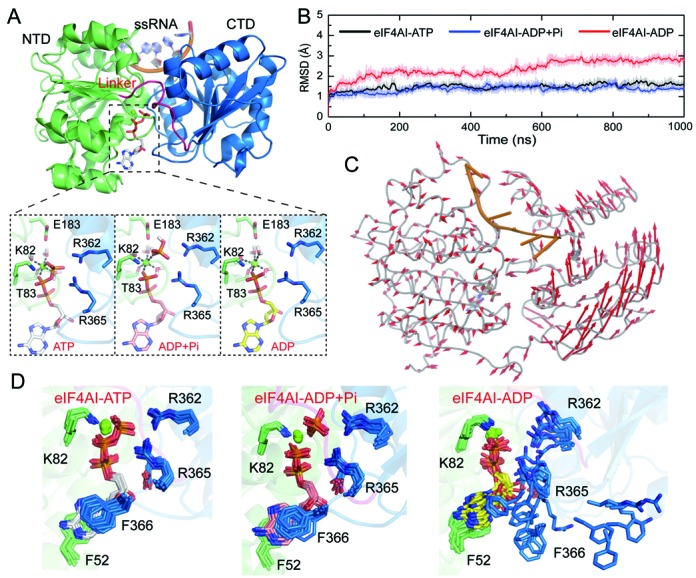
The cMD simulation of eIF4AI in different nucleotide-binding state. (**A**) Model of eIF4AI in the closed conformation. Residues from NTD, CTD and linker are shown in green, blue and magenta, respectively. (**B**) The time evolution of RMSD values of protein backbone atoms in cMD simulation. Structures from the cMD trajectories were aligned by their backbone atoms. (**C**) Collective motions corresponding to the first principle component, which accounts for 64.3% of the total movements, obtained by principal component analysis (PCA) on the simulation trajectory of the eIF4AI–ADP model. For PCA, structures were aligned by the backbone atoms of NTD, which were relatively stable during the cMD simulation. (**D**) Conformations of the nucleotide binding site residues during cMD simulations. For each model, snapshots were taken from the trajectory every 100 ns.

Among the different conformations of DBPs, the closed conformation induced by corporative binding of ATP and RNA is the most important one because both ATP hydrolysis and duplex unwinding are triggered from this conformational state. However, the closed conformation of eIF4AI in complex with ATP and RNA has not been determined by crystal structures, although its existence was verified by FRET experiments (36,37). Therefore, we built the structure of eIF4AI in the closed conformation by homology modeling based on the crystal structure of its close homolog eIF4AIII (PDBID: 2HYI) (23), which shares high sequence similarity with eIF4AI (Figure 1 and Supplementary Figure S1). To validate the modeled structure as well as to study the conformational changes of eIF4AI along the ATP hydrolysis cycle, we modeled the closed state eIF4AI in complex with ATP, ADP and ADP + Pi (Figure 1A) and performed 1 μs canonical molecular dynamic (cMD) simulations on each model. According to the simulation results, the closed state eIF4AI in complex with ATP and RNA or ADP + Pi and RNA remained stable during the 1 μs cMD simulation, as judged by the low root mean square deviation (RMSD) value (<1.5 Å) and small RMSD fluctuation (Figure 1B). In contrast, the eIF4AI-ADP model showed global conformational changes during the simulation as judged by a higher RMSD value (Figure 1B). Principle component analysis (PCA) (Figure 1C) also suggested a relative motion between the C-terminal domain (CTD) and N-terminal domain (NTD).

By assessing the structural stability of different regions, we found the NTD was stable in all three models but the inter-domain linker and CTD in the eIF4AI-ADP model underwent large conformational changes (Supplementary Figure S2A–B). In the ATP and ADP + Pi binding state, R365 and R362 from the conserved motif VI (HRIGRGGR) formed salt bridges with the γ-phosphate and residues involved in nucleotide binding remained stable during the entire simulation (Figure 1D). However, the nucleotide binding site residues, especially residues from the CTD, showed increased flexibility in the ADP binding state, which may be caused by decreased electrostatic interaction resulting from the absence of the γ-phosphate (Figure 1D and Supplementary Figure S2C). As the nucleotide acted as a molecular glue between NTD and CTD, the reduced interaction between ADP and CTD increased the tendency for the nucleotide binding cleft to open (Supplementary Figure S2C), eventually resulting in a global conformational change in the eIF4AI-ADP complex relative to the complexes of eIF4AI bound to either ATP or ADP + Pi. In crystal structures of eIF4A with different conformational states, the linker and CTD, especially regions involved in nucleotide binding, showed more structural flexibility than the NTD (Supplementary Figure S3). This is in accordance with our simulation results that CTD and linker conformations are more sensitive to alterations of the nucleotide state. The global conformational change in the ADP-binding state also influenced the stability of the RNA strand and conserved motifs involved in RNA binding (Supplementary Figure S4). The conformational changes in the RNA binding site and the linker region may also reflect the different sensitivity of protease cleavage sites near the RNA binding site and on the linker, when eIF4AI binds to ATP+RNA or ADP+RNA (38). As suggested by previous experimental studies, the ADP-binding state, which emerges after hydrolysis and Pi release, correlated with the reopening of the helicase core and release of the second RNA strand (10). Our simulation results recaptured this trend in the eIF4AI-ADP model, although we did not observe the complete opening of the nucleotide binding cleft and release of the RNA strand owning to the limitation in the accessible simulation time.

### Transient conformational change of the linker opens a backdoor channel that promotes phosphate release

**Figure 2.**
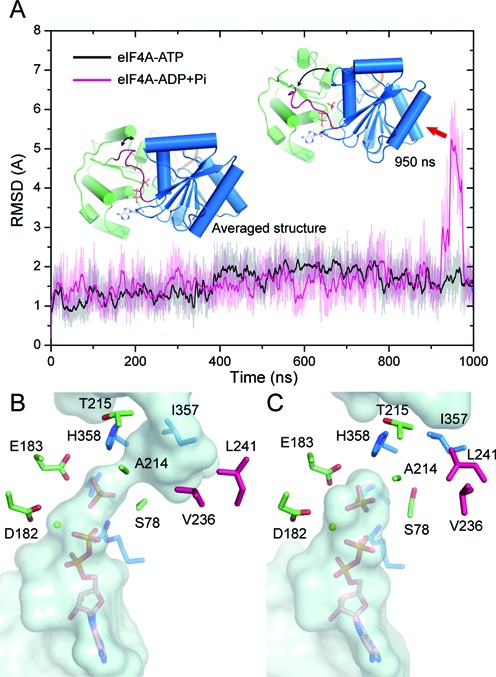
Linker dynamics of eIF4AI in the ATP and ADP-Pi binding states. (**A**) The time evolution of the root mean square deviation (RMSD) value of the linker backbone atoms in cMD simulation of the eIF4AI-ATP and eIF4AI-ADP+Pi model. Structures were aligned by the backbone atoms of the whole protein and RMSD values were calculated for the linker backbone atoms. The averaged structure and structure at 950ns in the trajectory of eIF4AI-ADP+Pi model, which displays the transient conformational change of the linker, are shown. (**B**) Surface representation of the backdoor channel. The residues around the channel are shown as sticks. The protein structure is taken from the MD snap at 950 ns. (**C**) In the most populated structure of the MD trajectory of the eIF4AI-ADP+Pi model, the backdoor channel cannot be observed.

Although the overall closed conformation remained stable in the ADP-Pi binding state, we noticed a significant conformational fluctuation of the inter-domain linker during the cMD simulation of the eIF4AI-ADP+Pi model (Figure 2A). This conformational fluctuation around 950 ns led to a transient separation between the linker and other parts of the protein (Figure 2A and Supplementary Movie S1) and we found the dissociation of the linker from the NTD-CTD interface created a channel that connected the γ-phosphate-binding site and protein surface (Figure 2B), which resembled the backdoor Pi release channel of ATPase motor protein, myosin (21,39,40). On the other hand, the linker bound stably to the NTD-CTD interface during the entire simulation of the eIF4AI-ATP model and the majority of the eIF4AI−ADP+Pi model with the backdoor channel closed by the interactions between several residues from NTD, CTD and linker region (Figure 2C). As the next step after the ADP-Pi binding state in the catalytic cycle of eIF4AI is Pi release, this transient opening of the backdoor channel may represent a ‘ready to release’ state while the majority of the conformations observed in the cMD trajectory represents the ‘Pi withholding’ state during the catalytic cycle of eIF4AI. We noticed that this channel observed in the cMD trajectory of the eIF4AI−ADP+Pi model was still too narrow for a Pi molecule. However, as the linker is intrinsically flexible, the opening of this channel could be further induced by the motion of Pi on its way out of the channel.

**Figure 3.**
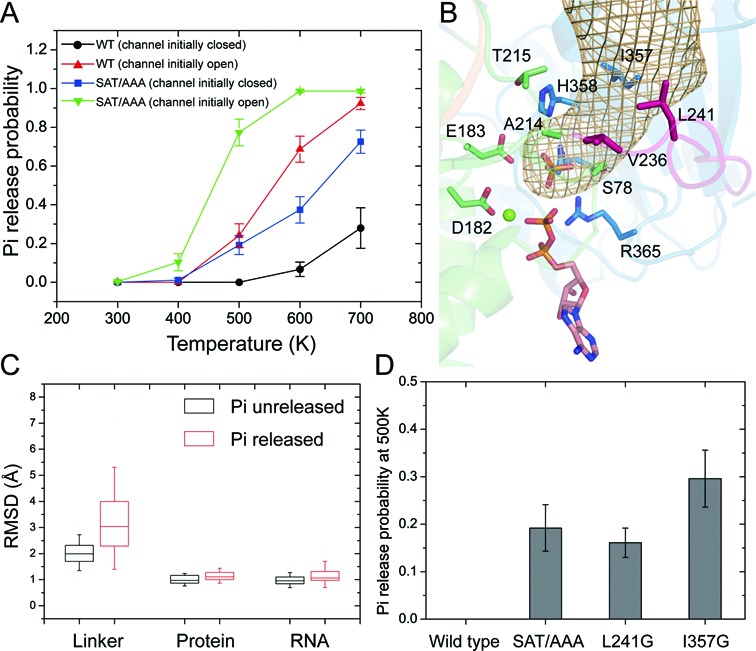
Simulation of Pi release using LES approach. (**A**) The probability of Pi release from wild type eIF4AI (WT) and eIF4AI with SAT/AAA mutation (SAT/AAA) at different temperatures. Simulations were starting from the conformations in which the channel was open or closed. All parts of the system were allowed to move during the simulations. The error bars represent the standard error of mean (SEM). (**B**) The Pi release pathway is shown as a mesh surface enclosing the space that highly occupied by the phosphorus atom (occupancy rate > 0.1) in the trajectories of Pi successfully escaping from hydrolysis site (calculated by VolMap in VMD). Residues around the Pi release channel are shown as sticks. (**C**) Box plot showing the distribution of RMSD values of the backbone atoms of the linker and the whole protein and heavy atoms of RNA in LES trajectories with or without Pi release. The RMSD value was calculated between structure from the final frame in each trajectory and the starting structure. LES trajectory was defined as ‘Pi released’ trajectory when at least one Pi replicate escaped from the hydrolysis site. (**D**) Pi release probability from wild type eIF4AI and several mutants at 500 K. The error bars represent SEM.

To test whether the opening of the backdoor channel, which results from the conformational change of the inter-domain linker, is related to Pi release, we simulated the Pi release form eIF4AI conformation with and without the existence of the backdoor channel, which represents the ‘ready to release’ and ‘Pi withholding’ states, respectively. As the timescale of the actual Pi release is on the order of milli-seconds or longer, which is beyond the time scale of cMD simulation, we adopted an approach similar to the one used by Martin Karplus *et al*. in simulating Pi release from myosin (21). The implementation of this approach in the Amber package is called locally enhanced sampling (LES) (33). In LES, the ligands (Pi in the present study) can be kept at a high temperature to speed up the crossover of energy barriers while the protein is kept at 300 K to avoid unrealistic motions (41). The detailed simulation procedure is described in Materials and Methods. Figure 3A shows the probabilities of Pi release from eIF4AI as a function of temperature. In the channel open state, Pi molecules started to be released at 500 K and 90% of Pi molecules escaped from the hydrolysis site at 700 K (red line). In contrast, only a small fraction of Pi molecules (∼30%) escaped from the hydrolysis site at 700 K (black line) in the channel closed state. By calculating the space occupancy of Pi molecules in the trajectories that all Pi replicates escaped from the hydrolysis site, we found the Pi release pathways observed in the LES simulations starting from the ‘channel open’ state or the ‘channel closed’ state overlapped with the backdoor channel observed in the cMD simulation (Figure 3B, Supplementary Movie S2). In addition, through analyzing protein structures in LES trajectories with and without Pi release, we found Pi release was accompanied by further movement of the linker while the overall protein and RNA structure remained stable (Figure 3C). From the movie that shows a typical Pi release from eIF4AI in the channel closed state, a significant conformational change of the linker can be observed before Pi escaping (Supplementary Movie S2). Therefore, these results suggest that the conformational change of the linker and opening of the backdoor channel indeed promote Pi release from eIF4AI in the ADP-Pi bound state.

### Mutations of the conserved SAT motif increased the linker flexibility and facilitated Pi release

**Figure 4.**
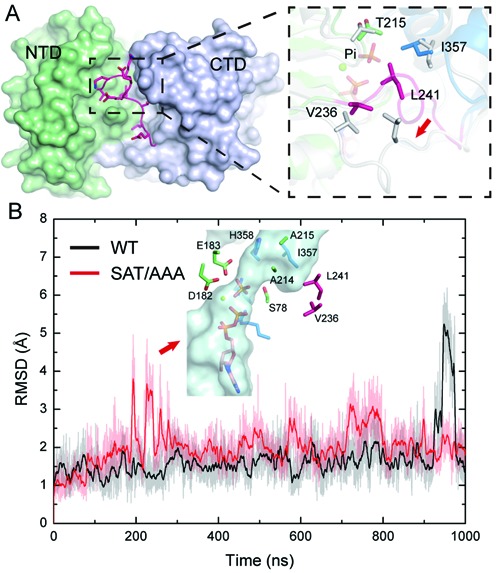
SAT/AAA mutation increases linker conformational flexibility and promotes the opening of the backdoor channel. (**A**) The linker binds to the NTD-CTD interface and forms a hydrophobic core with NTD and CTD residues. The residues involved in the hydrophobic core are shown as sticks. In the channel-closed state, the residues from NTD, linker and CTD are colored in green, magenta and blue, respectively. In the channel open state, all residues are colored in white. (**B**) The time evolution of the linker backbone atom RMSD value in the cMD simulation of eIF4AI-ADP+Pi and eIF4AI^SAT/AAA^-ADP+Pi models. Structures were aligned by the backbone atoms of the whole protein and RMSD values were calculated for the linker backbone atoms. A representative structure of the backdoor channel observed during the cMD simulation of the eIF4AI^SAT/AAA^-ADP+Pi model is also shown.

Pi release has been considered an important step in the functional cycles of several ATPase motor proteins, such as F1-ATPase, kinesin and myosin (21,42,43). Previous kinetic studies revealed that the post-hydrolysis ADP-Pi binding state of two DBPs—DbpA and Mss116p—bound more tightly to RNA than the ATP-bound state during their catalytic cycles, therefore being a good candidate for the actual work production state (15–17). Our cMD simulation results also suggested that the closed conformation of eIF4AI, which is critical to RNA unwinding, could be maintained in both ATP and ADP-Pi binding states but not in the ADP binding state. Therefore, we suspect factors that affect the backdoor channel may interfere with the ‘Pi withholding’ state and the coupling between ATPase and helicase. By comparing the MD snaps with open and closed backdoor channel (Figure 2B and C), we identified a hydrophobic core formed by four residues – T251, I357, V236 and L241, might play a critical role in gating this channel (Figure 4A). As V236 and L241 are located on the linker, the conformational fluctuation of the linker disrupted this hydrophobic packing, leading to the opening of the backdoor channel (Figure 4A). In addition to V236 and L241, another residue, T251, is also involved in the hydrophobic packing. Interestingly, T251 is part of the conserved motif III (SAT motif) of DEAD-box helicases. The SAT motif has been considered a key motif in the coupling between hydrolysis and unwinding, and mutation of SAT to AAA in eIF4AI was found to increase its ATPase activity while abolishing its helicase activity (12).

To determine whether the SAT to AAA mutation affects the opening of the backdoor channel and Pi release, we built a model of eIF4AI containing the SAT/AAA mutation in the ADP-Pi binding state and performed 1 μs cMD simulation. We found the SAT to AAA mutation itself did not directly open the back door channel since the channel was not observed in the starting structure (Supplementary Figure S5A). However, this mutation increased the frequency of conformational fluctuation during cMD simulation (Figure 4B), suggesting that the hydrophobic core may not only gate the backdoor channel but also contribute to the stable binding of the linker to the NTD-CTD interface. Similarly, this conformational fluctuation of the linker also opens the backdoor channel in the eIF4AI^SAT/AAA^-ADP+Pi model (Figure 4B). In addition, the channel bottleneck radius and channel volume were also increased by SAT/AAA mutation (Supplementary Figure S5B and SC). On the other hand, the overall stability of eIF4AI^SAT/AAA^-ADP+Pi model is very similar to that of the eIF4AI-ADP+Pi model and nucleotide binding site residues also remained stable (Supplementary Figure S5D and SE). This is expected since the complete hydrolysis site exists only in the closed conformation and the SAT mutation does not impair hydrolysis in eIF4AI.

LES simulation results also indicated that the conformational change of the linker facilitated the Pi release from the backdoor channel in eIF4AI^SAT/AAA^ model (Figure 3A, Supplementary Figure S6A and Supplementary Movie S3) and was accompanied by further movement of the linker (Supplementary Figure S6B). The barrier for Pi release is lowered by the SAT/AAA mutation even in the ‘Pi withholding’ state (Figure 3A, blue line compared with black line), which may due to the fact that SAT/AAA mutation destabilized the linker conformation (Figure 2A) and the dissociation of the linker from the NTD-CTD interface was required for the opening of the backdoor channel. If the backdoor channel was kept closed by fixing the linker conformation, no Pi release from wild type eIF4AI was be observed and the Pi release probability from the SAT/AAA mutant was also reduced (Supplementary Figure S6C). *In silico* mutations of two other hydrophobic residues taking part in gating the backdoor channel – L241 and I357 were also found to increase the probability of Pi release from eIF4AI in the ‘Pi withholding’ state at 500 K, the highest temperature before Pi release from the wild type protein (Figure 3D). Taken together, these simulation results suggested that the SAT motif and dynamics of the inter-domain linker in eIF4AI play a critical role in regulating Pi release in the post-hydrolysis state and may thus take part in the coupling between ATP hydrolysis and unwinding.

### Mutation of the gating residues in the backdoor channel enhanced ATP hydrolysis but impaired RNA unwinding

**Figure 5.**
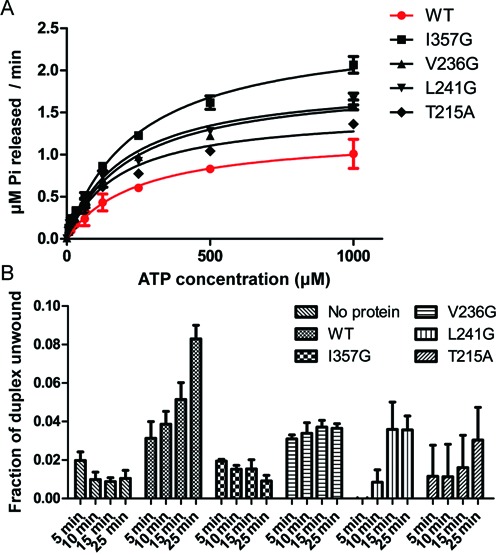
Mutational studies of the gating residues in the backdoor Pi release channel. (**A**) ATPase activity of recombinant 6×His-eIF4AI (N-terminally His-tagged) under saturating RNA concentration (250 μg/ml). The enzyme concentration was 0.4 μM. (**B**) RNA helicase activity of recombinant 6×His-eIF4AI under saturating ATP concentration (2 mM). The error bar represents SEM (n = 3).

Previous studies suggest the Pi release step is the rate-limiting step of DBPs hydrolysis cycles (15–17), and as such, accelerating Pi release will increase the enzyme turnover rate. However, fast Pi release may also shorten the time DBPs reside in the closed conformational state therefore impairing unwinding activity. This may explain the observed decoupling effect of the SAT mutation in eIF4AI. To test this hypothesis, we mutated four residues forming the hydrophobic core that gates the Pi release channel, including T215, I357, V236 and L241, to less hydrophobic ones and evaluated their ATP hydrolysis activity and RNA unwinding activity. Similar to the reported enzymatic activity of eIF4AI^SAT/AAA^ (12), all of the mutations increased ATPase activity (Figure 5A and Supplementary Figure S7A) but decreased RNA unwinding (Figure 5B and Supplementary Figure S7B). The mutation of the most bulky and hydrophobic residue – I357, which would have the greatest impact on the hydrophobic packing and channel size, increased the K_cat_ value by 2-fold while had little impact on the K_m_ value, indicating that the enzyme turn-over was accelerated (Table 1). Accordingly, the I357G mutant had the lowest unwinding activity (Figure 5B). These changes in enzymatic activity were not results from the alterations of global protein folding states, as revealed by the circular dichroism experiments (Supplementary Figure S7C). In addition, we found the presence of excess Pi in the reaction buffer did not affect the helicase activity of eIF4AI (Supplementary Figure S7D), which is in accordance with previous kinetic studies that phosphate release from DDX may be irreversible because Pi rebinding is slow and weak (15). These results suggest the hydrophobic core that acts as a gate in the backdoor Pi release channel indeed has an important role in the coupling between ATP hydrolysis and RNA unwinding.

**Table 1.** Michaelis-Menten kinetic parameters of ATP hydrolysis derived from the curves shown in Figure 5A

	WT	I357G	V236G	L241G	T215A
**k_cat_(min^−1^)**	3.1 ± 0.3	6.3 ± 0.2	4.7 ± 0.3	4.7 ± 0.3	3.8 ± 0.2
**Km(μM)**	240 ± 50	250 ± 20	220 ± 30	200 ± 30	180 ± 30

### Regulation of eIF4AI's activity by other inter-domain linker residues

**Figure 6.**
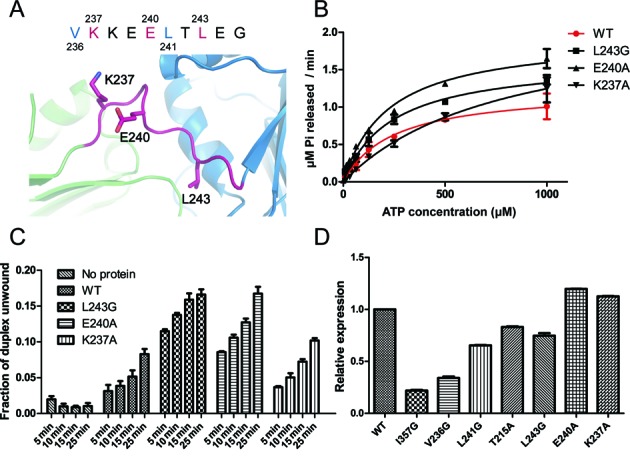
Functions of additional linker residues in regulating the activity of eIF4AI. (**A**) Linker residues that face solvent and do not take part in the hydrophobic core. (**B**) ATPase activity of eIF4AI mutants under saturating RNA concentration (250 μg/ml). The enzyme concentration was 0.4 μM. (**C**) RNA helicase activity of eIF4AI mutants under saturating ATP concentration (2 mM). (**D**) *In vitro* translation efficiencies of WT and mutant eIF4AI. The error bar represents SEM (n = 3).

The aforementioned simulation and experimental results suggest an important role of the inter-domain linker in the regulation of eIF4AI's activity. However, compared with other conserved motifs, this linker has attracted less attention. A previous study reported mutations of two polar residues in the linker – K237 and E240, which are located next to V236 and L241, respectively (Figure 6A), also increased eIF4AI's ATPase activity. But the unwinding activities of those mutants were not determined (13). We then performed mutational studies on these two residues as well to characterize the role of the linker in coupling the ATPase to the helicase activity. In addition, we mutated another hydrophobic residue on the linker, L243, which does not take part in forming the hydrophobic core. Unexpectedly, although E240A and L243G increased ATPase activities as other linker mutations did (Figure 6B, Table 2 and Supplementary Figure S7E), the unwinding activity of eIF4AI was also increased by those two mutations (Figure 6C and Supplementary Figure S7F). On the other hand, eIF4AI mutant with K237A showed a similar helicase activity compared with wild type (Figure 6C and Supplementary Figure S7F).

**Table 2.** Michaelis-Menten kinetic parameters of ATP hydrolysis derived from the curves shown in Figure 6B

	WT	L243G	E240A	K237A
**k_cat_(min^−1^)**	3.1 ± 0.3	4.1 ± 0.2	4.9 ± 0.2	5.6 ± 0.7
**K_m_ (μM)**	240 ± 50	240 ± 40	220 ± 30	790 ± 190

We further examined the impact of all these mutations, including mutations that simultaneously enhanced ATPase and helicase activity as well as the decoupling mutations, on the translation initiation activity eIF4AI. As shown in Figure 6D, except for E240A and K237A, all mutations decreased the *in vitro* translation activity of eIF4AI. It is conceivable that the mutations with defects in unwinding, such as I357G, will impair translation activity. But L243G, which significantly enhanced RNA unwinding, was also found to decrease the translation initiation activity (Figure 6D). In addition to its intrinsic ATPase and RNA helicase activity, the effects of eIF4AI on translation initiation also depends on its interaction with other initiation factors including eIF4E and eIF4G (44). To reconcile the apparent discrepancy between the enhanced ATPase and RNA helicase activities of the L243G mutant and the decreased translation initiation, we investigated the interaction between those mutants and eIF4E/4G. Indeed, we observed a decrease in the interaction between L243G mutant and eIF4E and eIF4G (Supplementary Figure S8). These results revealed a complex role of the inter-domain linker in fine-tuning the enzymatic activity and translation activity of eIF4AI.

## DISCUSSION

In this study, we adopted a simulation-driven approach to study the structural and functional dynamics of eIF4AI – the founding member of DEAD-box family of helicases. Our simulation and experimental results revealed that the inter-domain linker of eIF4AI plays a critical role in the coupling of ATP hydrolysis and RNA unwinding through regulating Pi release from the ATP-binding site. The linker interacts with both the NTD and CTD in the closed conformation, and the interaction is stabilized by hydrophobic packing among residues from the linker, NTD and CTD. Pi release after ATP hydrolysis is retarded by this hydrophobic core as suggested by LES simulation. The mutation of the SAT motif (SAT to AAA), which was reported to decouple ATP hydrolysis and RNA unwinding by eIF4AI, destabilized the linker-NTD-CTD interface and increased the probabilities of Pi release. These results suggested that the hydrophobic core might be involved in the coupling of the ATPase and the helicase activities of eIF4AI. The *in silico* model was validated by mutational studies. Besides SAT residues, destabilizing the hydrophobic packing at the tripartite interface through mutagenesis of other residues also led to the decoupling between ATP hydrolysis and RNA unwinding. Together with previous experimental findings that Pi release is the rate-limiting step in the catalytic cycles of DBPs and the unwinding events take place in the ADP-Pi state (15–17), we suggest that a stable linker-NTD-CTD interface in the closed conformation ensures the coupling between hydrolysis and unwinding by preventing Pi leaking before strand separation.

A similar Pi gating mechanism has been demonstrated for other ATPase motor proteins. In free myosin, Pi release is blocked by several residues in a backdoor channel in order to ensure the actin-binding step takes place before Pi escape (21). Kinesin also utilizes a Pi gating mechanism to acquire processivity (43). From available structures of DBPs in the closed conformational state, it can be seen that despite the diversity in linker sequences, the linker-NTD-CTD interface found in eIF4A appear to exist in all members of the DBP family, although the tripartite interface are formed by different types of interactions (Supplementary Figure S9). This observation raises the possibility that the linkers in other DBPs also take part in gating the Pi release after hydrolysis and therefore regulating ATPase-helicase coupling. However, the non-hydrolizable ATP analogue ADP-BeFx was found to promote duplex unwinding of several DBPs (14). This discrepancy may be attributable to the fact that the ability to bind RNA and maintain the closed state is different among DBPs. Many DBPs have non-conserved N-terminal or C-terminal extensions, which provide additional RNA binding sites. For DBPs, which tightly bind to RNA and have strong helicase activity, the closed conformation merely induced by nucleotide binding may be enough for duplex unwinding. Indeed, even AMPPNP, a non-hydrolysable ATP analog which can not promote duplex unwinding of most DBPs, was found to promote duplex unwinding in four cases (45). But for weak DEAD-box helicases such as eIF4AI, the post-hydrolysis ADP-Pi binding state may be required to extend the ‘duty time’. Accordingly, the unwinding in presence of ADP-BeFx is far less efficient than ATP, indicating ADP-BeFx cannot mimic all the states of ATP that promote unwinding (14). As the unwinding activities of different DBPs largely depended on the properties of the individual proteins and their substrates, whether gating Pi release through the inter-domain linker is a common theme in all DBPs and whether this Pi gating mechanism correlates with their unwinding activities will have to await further experimental characterizations.

Our simulation and mutagenesis studies also indicated the inter-domain linker plays important and diverse roles in fine-tuning eIF4A's enzymatic activity and *in vivo* functions. On the one hand, two residues (V236 and L241) from the linker participate in forming the hydrophobic gate to slow Pi release before duplex unwinding and the movement of the highly polar Pi molecule may in turn weaken the hydrophobic interaction, therefore enabling Pi release and entering the next catalytic cycle. As the linker is more flexible than NTD and CTD, the movement of this linker may control the opening and closing of the Pi release channel, thus maintaining the balance between unwinding and recycling. On the other hand, we found several other residues on the linker, including K237, E240 and L243, play an auto-inhibitory role of eIF4AI's enzymatic activity. These three residues may interfere with eIF4AI's enzymatic activity through destabilizing the closed conformation or by decreasing the probability of eIF4AI to access the closed conformation, as we observed elevation of both ATPase and helicase activities when these residues were mutated. It is noteworthy that the trend of the changes in enzymatic activity did not always agree with the change in eIF4AI's activity in translation initiation. Although L243G increased both ATPase activity and helicase activity, it inhibited the *in vitro* translation initiation activity of eIF4AI. *In vivo* pull-down experiments showed the L243G mutation decreased the interaction between eIF4AI and eIF4G and eIF4E, two other components of eIF4F, offering a plausible explanation of the discrepancy. While the binding interface between eIF4AI and eIF4E is not known, the crystal structure of the yeast eIF4A-eIF4G complex indicated that the linker does not directly interact with middle domain of eIF4G (46). There's possibility that the unsolved C-terminal domain of eIF4G that carries a second eIF4AI binding site interacts with the linker. The inhibition between eIF4AI and eIF4G by L243G may also be mediated through an allosteric mechanism, which is reminiscent of a specific inhibitor of eIF4AI, pateamine A, that enhances the enzymatic activity of eIF4A while disrupting the integrity of eIF4F (4,47). All these observations suggested a critical and complex role of the linker in regulating eIF4AI's enzymatic activity and *in vivo* function. Besides regulating Pi release, the linker may also function in controlling the global orientations of the two domains and may even directly take part in the interactions between eIF4AI and its cofactors. It would be an interesting topic to further explore whether the inter-domain linker act as a driving force in regulating the enzymatic and in vivo translation activity of eIF4AI. In addition, the linker sequences in different DBPs may have been optimized differently for specific *in vivo* functions since its sequence is not conserved among DBPs. Given its critical role in regulating the coupling of ATPase and helicase activities of eIF4A and its interaction with eIF4E/4G, the linker-NTD-CTD interface may be a promising new target site to achieve selective inhibition of eIF4AI's activity over other DPBs.

## Supplementary Material

SUPPLEMENTARY DATA
