# Supplementary material for: A gating mechanism for Pi release governs the mRNA unwinding by eIF4AI during translation initiation
Source: Nucleic Acids Res. 2015 Oct 12;43(21):10157–67. doi: 10.1093/nar/gkv1033 (PMC4666354; doi:10.1093/nar/gkv1033)
Supplement: SUPPLEMENTARY DATA [file supp_43_21_10157__index.html]

A gating mechanism for Pi release governs the mRNA unwinding by eIF4AI during translation initiation — SUPPLEMENTARY DATA 

# A gating mechanism for Pi release governs the mRNA unwinding by eIF4AI during translation initiation

## SUPPLEMENTARY DATA

- SUPPLEMENTARY DATA
- SUPPLEMENTARY DATA
- SUPPLEMENTARY DATA
- SUPPLEMENTARY DATA
