# Supplementary material for: A gating mechanism for Pi release governs the mRNA unwinding by eIF4AI during translation initiation
Source: Nucleic Acids Res. 2015 Oct 12;43(21):10157–67. doi: 10.1093/nar/gkv1033 (PMC4666354; doi:10.1093/nar/gkv1033)
Supplement: SUPPLEMENTARY DATA [file supp_gkv1033_nar-01694-v-2015-File010.pdf]

**Supplementary Information for**

# A gating mechanism for Pi release governs the mRNA unwinding by eIF4AI during translation initiation


Junyan Lu[a,b,1], Chenxiao Jiang[a,1], Xiaojing Li[a,1], Lizhi Jiang[a], Zengxia Li[a], Tilman Schneider-Poetsch[c], Jianwei Liu[d], Kunqian Yu[b], Jun O. Liu[e], Hualiang Jiang[b], Cheng Luo[b,2], Yongjun Dang[a,2]

[a]Key Laboratory of Metabolism and Molecular Medicine, the Ministry of Education, Department of Biochemistry and Molecular Biology, School of Basic Medical Sciences, Fudan University, Shanghai, 200032, China;

[b]Drug Discovery and Design Center, State Key Laboratory of Drug Research, Shanghai Institute of Materia Medica, Chinese Academy of Sciences, Shanghai 201203, China;

[c]Chemical Genetics Laboratory, RIKEN, Wako, Saitama, 351-0198, Japan;

[d]Department of Chemistry, Shanghai Key Lab of Chemical Biology for Protein Research & Institutes of Biomedical Sciences, Fudan University, Shanghai, 200433, China;

[e]Departments of Pharmacology and Oncology, Johns Hopkins University School of Medicine, Baltimore, MD, 21205, USA

# Supplementary Methods

***In vivo* pull-down assays.** HEK293T cells were cultured in DMEM high glucose media (Invitrogen Inc.) plus 10% fetal bovine serum (Invitrogen Inc.) and maintained in 5%$CO^2$ at 37$^OC$. pcDNA3.1/HisA-eIF4AI or mutants as indicated were transfected into HEK293T cells. After 48 hr, cells were lysed with lysis buffer [20 mM imidazole (pH 8.0), 150 mM NaCl, 3 mM KCl, 3 mM Na2HPO4, 5 mM KH2PO4 ,1% Triton X-100 and a cocktail of protease inhibitors (Sigma Aldrich)]. Cell lysate was subsequently incubated with Ni-NTA resin for 4 hr, and washed 4 times with same buffer. Final elutes were subjected to SDS-PAGE and western blotting.

# Supplementary Figures

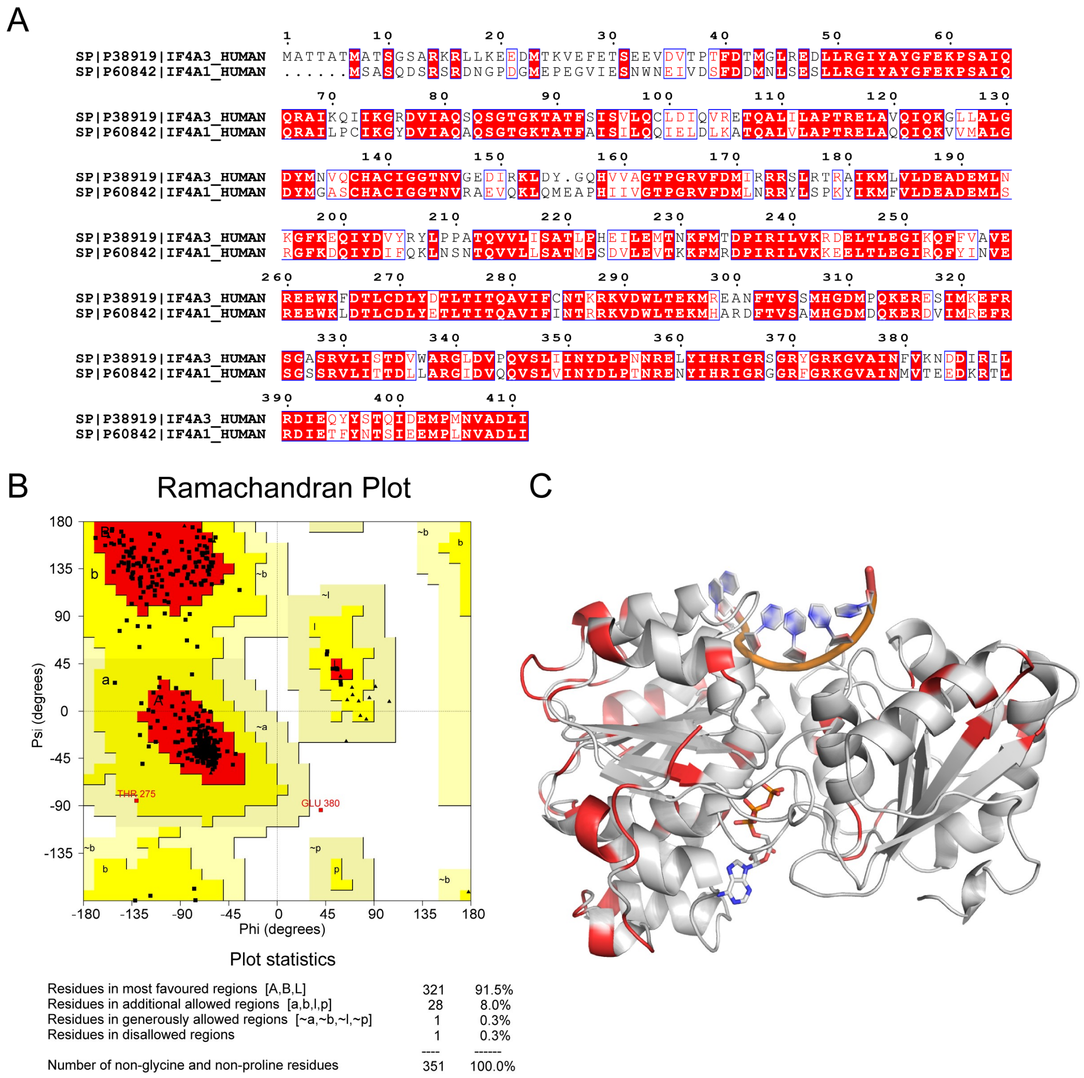


**Figure S1. Homology modeling of eIF4AI in closed conformation.** (A) Sequence alignment between eIF4AI and eIF4AIII. The sequence identity is over 65.7%. (B) Ramachandran plot of the eIF4AI homology model. (C) The non-conserved residues between eIF4AI and eIF4AIII are shown in red. Most of these non-conserved residues are located on the surface of the protein and do not take part in ATP or RNA binding.

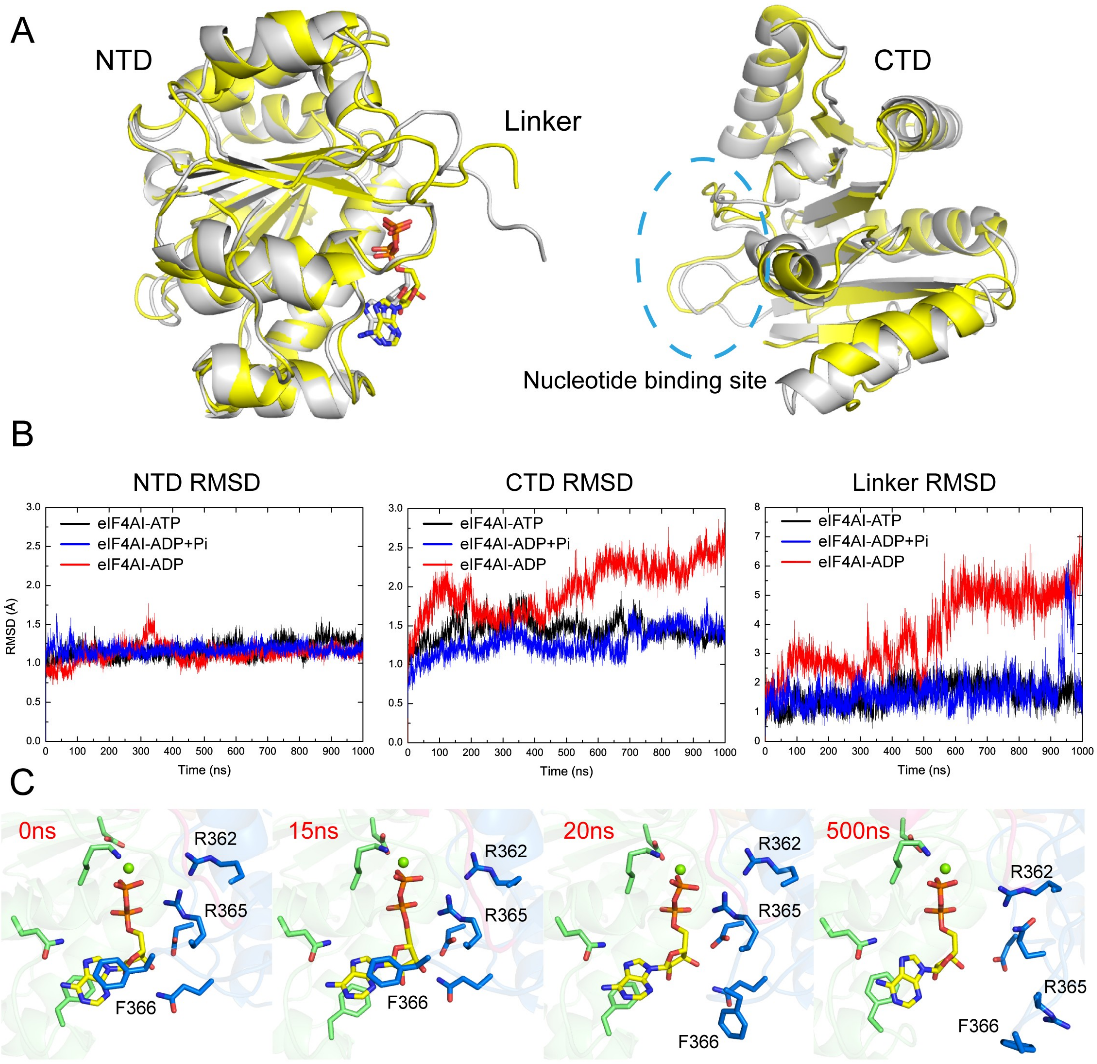


**Figure S2. Canonical Molecular dynamic simulations of closed eIF4AI in complex with ATP and ADP.** (A) Superimposition of the NTD (including the linker) and CTD structures. The starting structure of each model is shown in white and the structure taken from the last frame is shown in yellow. (B) The evolution of the protein backbone atom RMSD value from NTD, CTD and the linker in MD simulations. (C) Snapshots that show the critical changes in the nucleotide binding site residues during the simulation trajectory of the eIF4AI-ADP model.

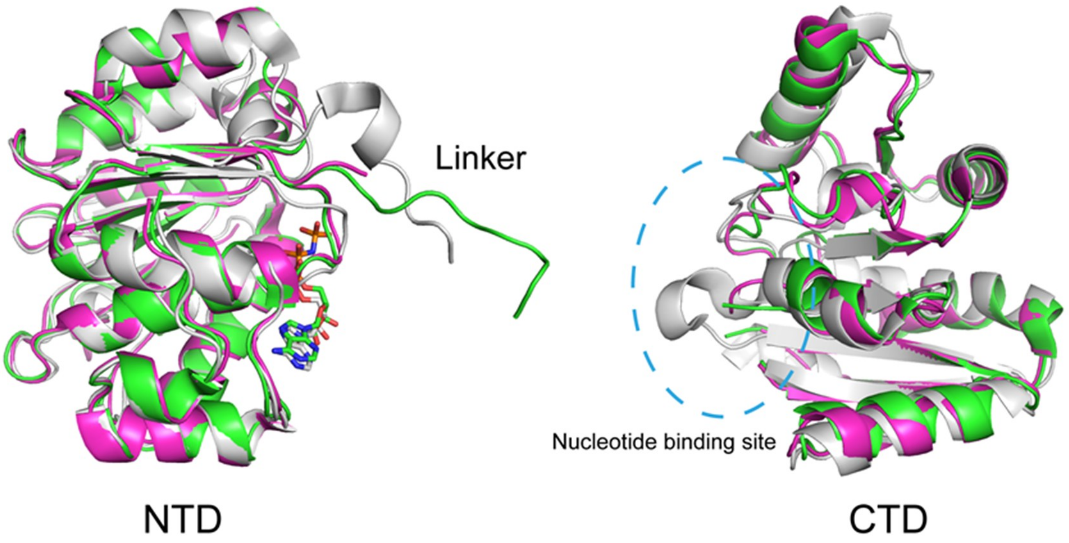


**Figure S3. Superimposition of eIF4A crystal structures in different conformational states.** The NTD and CTD structures in white are from the crystal structure of the closed state eIF4AIII in the EJC complex (PDBID: 2HYI). The NTD and CTD structure in green are from the crystal structure of yeast eIF4A bound to eIF4G (semi-open state) (PDBID:2VSO). The structures of the NTD and CTD in the open state are shown in magenta, and they are from the human eIF4AI NTD crystal structure (PDBID: 2G9N) and yeast eIF4A CTD crystal structure (PDBID: 1FUK). Some residues in the nucleotide binding sites of the CTD are missing from crystal structures in the open and semi-open state, indicating these regions are flexible.

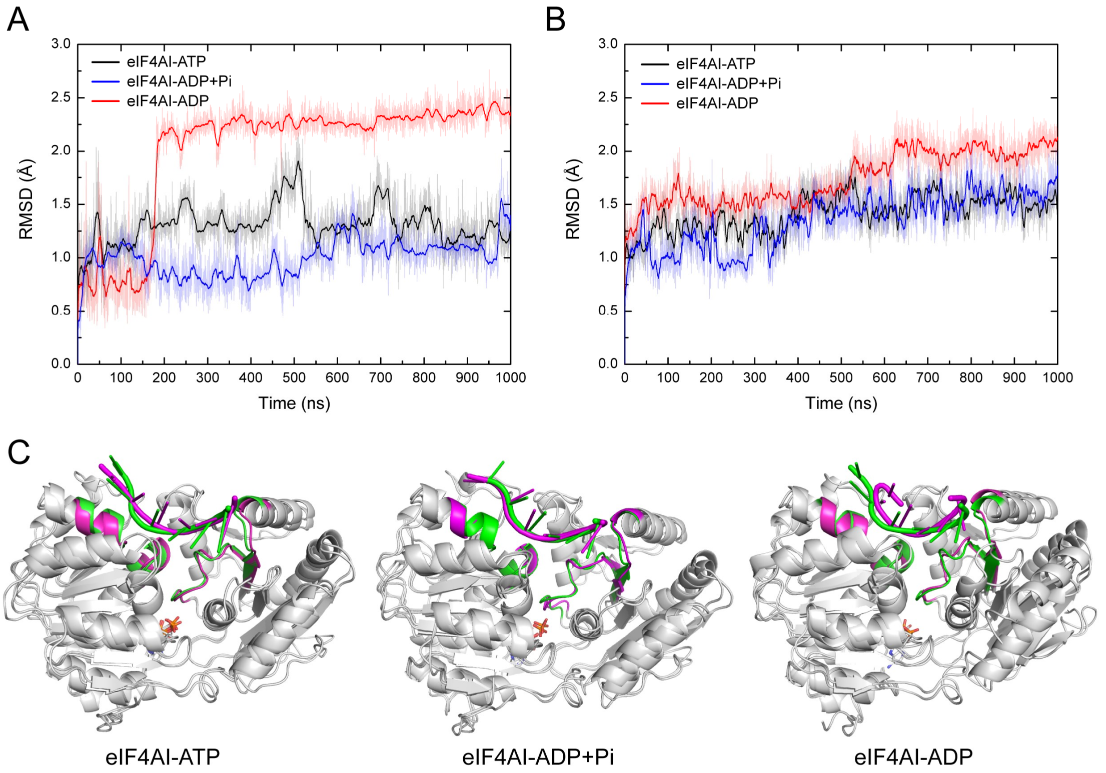


**Figure S4. Structural changes of ssRNA and RNA binding site in MD simulations.** (A) The RMSD evolution of heavy atoms in ssRNA during MD simulations. (B) The RMSD evolution of heavy atoms in conserved motifs involved in RNA binding (motif Ia, Ib, IV and V) during MD simulations. (C) Superimposition of the starting structure (green) and structure from the final frame (magenta) of the MD trajectory in each model.

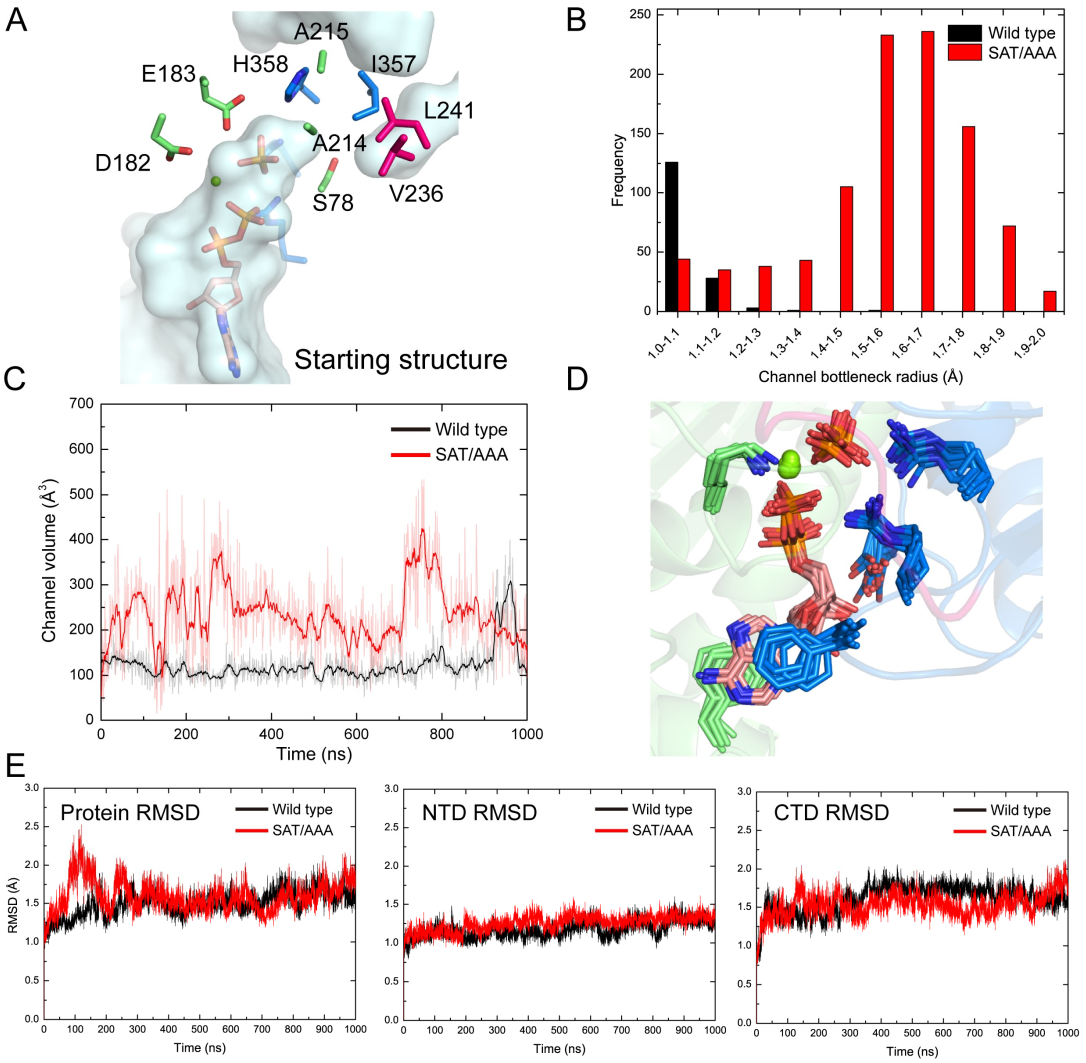


**Figure S5. cMD simulation of the eIF4AI$^{SAT/AAA}$-ADP+Pi model.** (A) Surface representation of the backdoor channel in the starting structure of the eIF4AI$^{SAT/AAA}$-ADP+Pi model. (B) Distribution of the bottleneck radius of the backdoor channel. Caver 3.0 was used to estimate the bottleneck radius and totally 1,000 snapshots from each MD trajectory were used. (C) The evolution of the backdoor channel volume. Fpocket was used to calculate the backdoor channel volume of the snapshots taken from MD trajectories. (D) Conformations of nucleotide binding site residues during MD simulation of the eIF4AI$^{SAT/AAA}$ model. Snapshots were taken from the MD trajectory every 100ns. (E) The evolution of RMSD values of the backbone atoms from the whole protein, NTD and CTD in cMD simulations of the eIF4AI$^{SAT/AAA}$-ADP+Pi model.

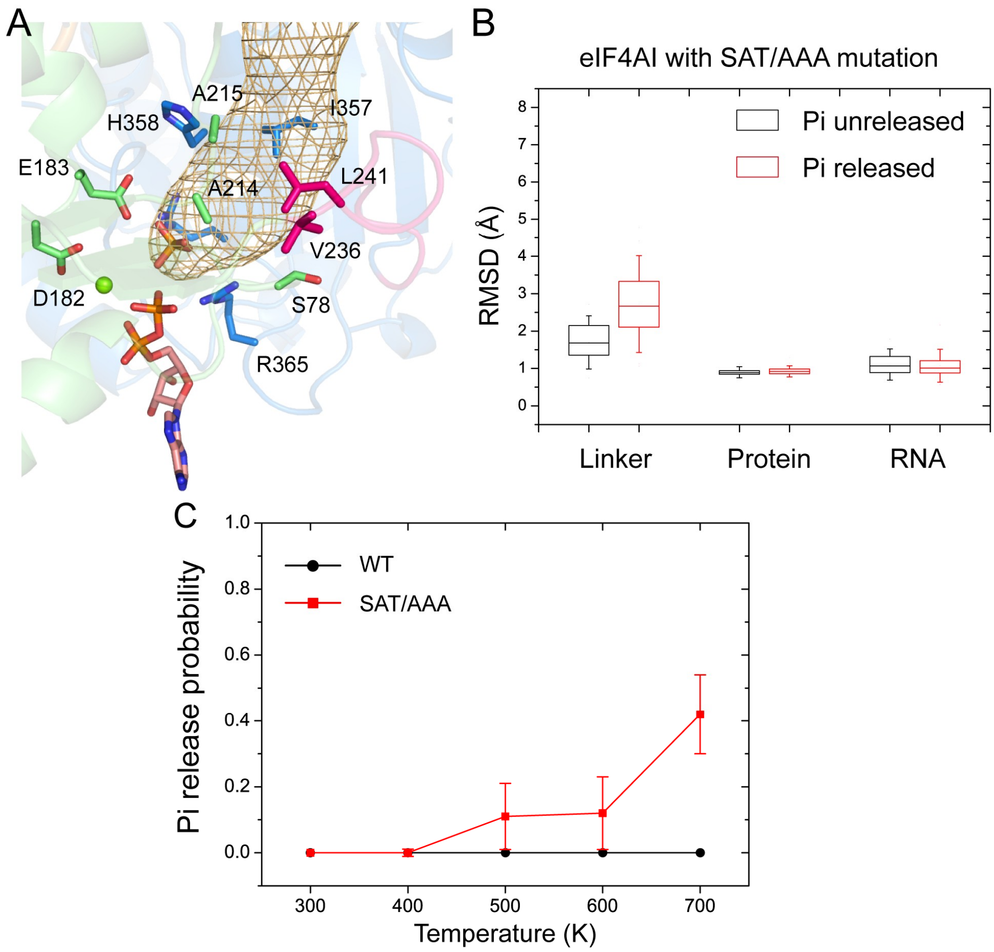


**Figure S6. Simulation of Pi release from eIF4AI with the SAT/AAA mutation.** (A) The detailed representation of the backdoor Pi release pathway in eIF4AI with the SAT/AAA mutation. Residues around the Pi release route are shown as sticks. The Pi release pathway is shown as a mesh surface enclosing the space that is highly occupied by the phosphorus atom (occupancy rate > 0.1) in the trajectories that Pi successfully escaped from the hydrolysis site (calculated by VolMap in VMD). (B) Box plot showing the distribution of backbone atom RMSD values of the linker and the whole protein and heavy atoms of RNA in all LES trajectories with or without Pi release. The RMSD value was calculated from the structure of the final frame in each trajectory compared to the starting structure. LES trajectory was defined as a “Pi released” trajectory when at least one Pi replicate escaped from the hydrolysis site. (C) The Pi release probabilities at different temperature when the backdoor channel was kept closed by fixing the linker conformation during the LES simulations.

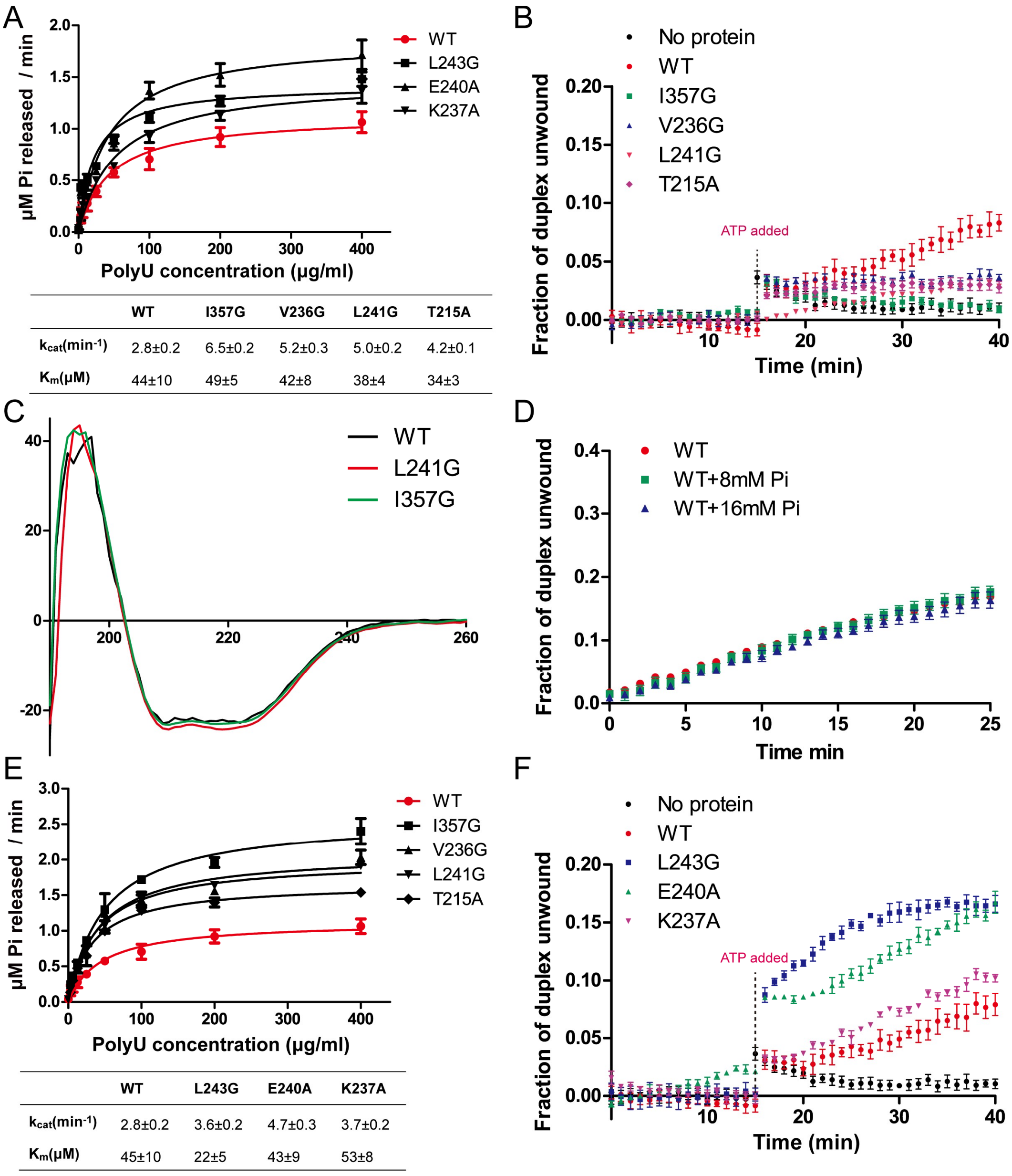


| | WT | I357G | V236G | L241G | T215A |
|---|---|---|---|---|---|
| $k_{cat}$(min$^{-1}$) | 2.8±0.2 | 6.5±0.2 | 5.2±0.3 | 5.0±0.2 | 4.2±0.1 |
| $K_m$(μM) | 44±10 | 49±5 | 42±8 | 38±4 | 34±3 |

| | WT | L243G | E240A | K237A |
|---|---|---|---|---|
| $k_{cat}$(min$^{-1}$) | 2.8±0.2 | 3.6±0.2 | 4.7±0.3 | 3.7±0.2 |
| $K_m$(μM) | 45±10 | 22±5 | 43±9 | 53±8 |

**Figure S7. ATPase and helicase activity assays for wild type or mutated eIF4AI.** (A and E) ATPase assays in the presence of saturating ATP (1 mM) and varying concentrations of polyU. The protein concentration in the reaction mixture was 0.4 μM. (B and F) The fraction of duplex unwound as a function of reaction time. 2 mM ATP was added to the reaction mixture at 15 min. Because the unwinding reaction started as soon as the ATP was added and the equipment operations took time, there were gaps between the florescent signals before and after 15 min. In addition, because of the systematic error of the detection equipment, the baseline florescent signals after ATP addition are higher than that before ATP addition. The concentrations of RNA duplex and protein in the helicase assay were 50nM and 1μM, respectively. The

ATPase and helicase assays were repeated three times at least and error bars represent SEM. (C) Circular dichroism experiment results of wild type eIF4AI, eIF4AI$^{L241G}$ and eIF4AI$^{I357G}$. Mutation of large hydrophobic residues into glycine possesses certain risks of altering the global protein folding states. To excluded this probability, circular dichroism experiments were performed on the wild type eIF4AI, I357G mutant and L241G mutant. I357 is on an alpha-helix region and therefore I357G is the most risky mutation. L241 and other hydrophobic residues we mutated are on the unstructured linker region. According to the results, their CD signals are almost the same, indicating the protein folding was not affected by these two mutations. (D) Helicase activity of wild type eIF4AI in the presence of excess Pi. The time ATP was added to the reaction mixture was defined as the starting time (0 min).

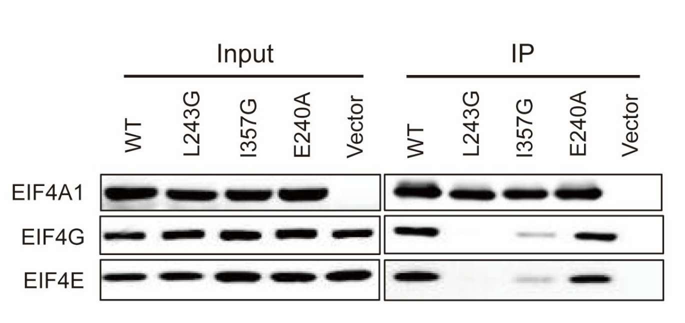


**Figure S8. The interaction of wild type eIF4AI$^{WT}$, eIF4AI$^{L243G}$ and eIF4AI$^{I357G}$ with eIF4E and eIF4G *in vivo*.** The indicated plasmids were transfected into HEK293T cells. The capability of eIF4AI and its mutants to form the eIF4F complex were detected by western blotting with antibodies against eIF4E, eIF4G and His tag after pulldown with NTA-Ni agarose.

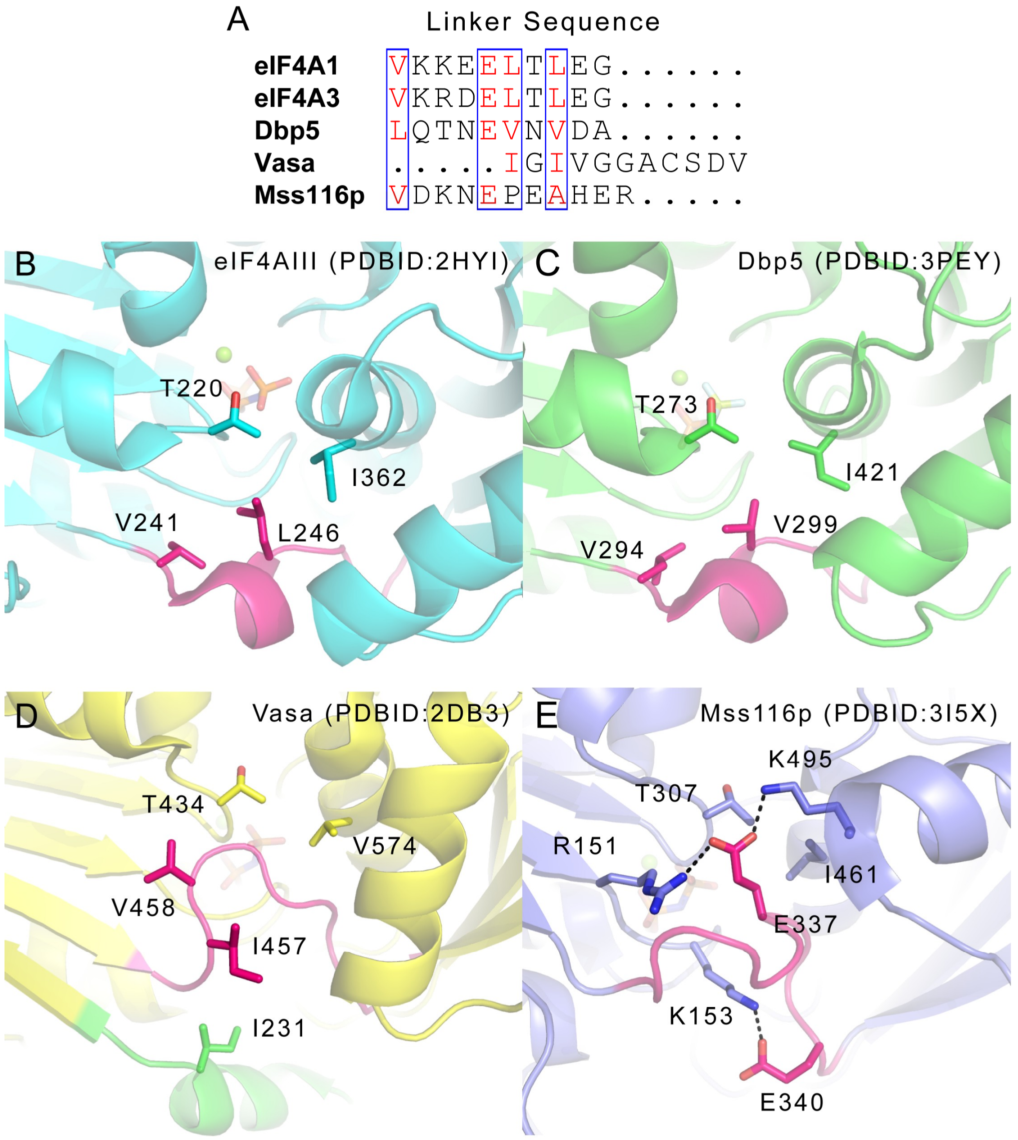


**Figure S9. Comparison of the sequences and conformations of the inter-domain linker in different crystal structures of closed state DBPs.** (A) Sequence alignment of the linkers from different DBPs indicates only the linker of eIF4AIII shares high similarity with the linker of eIF4AI. In closed state eIF4AIII and Dbp5, hydrophobic packing among residues from the linker, NTD and CTD can also be observed (D and E). In closed state Vasa, although the linker does not form hydrophobic packing with NTD-CTD interface, its binding to the NTD-CTD interface may be stabilized by the NTD extension (D). In closed state Mss116p, the linker interacts with NTD and CTD mainly through polar interactions (E).

# Supplementary Table

**Table S1. Primer lists for eIF4AI mutations**

| **Primer lists** | |
|---|---|
| eIF4AI-T215A-F | TTT TGCTGT CAG CCG CAA TGC CTT CTG ATG TGC TTG AG |
| eIF4AI-T215A-R | ACA TCA GAA GGC ATT GCG GCT GAC AGC AAA ACT ACC TG |
| eIF4AI-V236G-F | CCA TTC GGA TTC TTG GCA AGA AGG AAG AGT TGA CC |
| eIF4AI-V236G-R | AAC TCT TCC TTC TTG CCA AGA ATC CGA ATG GGG TC |
| eIF4AI-K237A-F | ATT CGG ATT CTT GTC GCG AAG GAA GAG TTG ACC CTG |
| eIF4AI-K237A-R | GTC AAC TCT TCC TTC GCG ACA AGA ATC CGA ATG G |
| eIF4AI-E240A -F | GTC AAG AAG GAA GCG TTG ACC CTG GAG GGT ATC C |
| eIF4AI-E240A-R | CTC CAG GGT CAA CGC TTC CTT CTT GAC AAG AAT CC |
| eIF4AI-L241G-F | TCA AGA AGG AA AGG GGA CCC TGG AGG GTA TCC GCC AGT TC |
| eIF4AI-L241G-R | ATA CCC TCC AGG GTC CCC TCT TCC TTC TTG ACA AGA ATC CG |
| eIF4AI-L243G-F | AGAAGGAAGAGTTGACCGGCGAGGGTATCCGCC AGTTC |
| eIF4AI-L243G-R | AACTGGCGGATACCCTCGCCGGTCAACTCTTCCTT CTT |
| eIF4AI-I357G-F | AAC AGG GAA AAC TAT GGC CAC AGA ATC GGT CGA GG |
| eIF4AI-I357G-R | TCG ACC GAT TCT GTG GCC ATA GTT TTC CCT GTT GG |

## Legends for Supplementary Movies

**Movie S1. The conformational fluctuation of the inter-domain linker in MD trajectory of eIF4AI-ADP+Pi model.** The protein and RNA are shown as cartoons with NTD, linker, CTD and RNA colored in green, red, blue and orange, respectively. For better presentation, the trajectory is smoothed with a window size of 10 frames.

**Movie S2. Pi release from the backdoor channel in wild type eIF4AI.** The movie shows a typical Pi release trajectory from the wild type eIF4AI. The protein and RNA are shown as cartoons with NTD, linker, CTD and RNA colored in green, red, blue and orange, respectively. ADP and residues forming the hydrophobic core are shown as sticks. Pi is shown as a yellow sphere. For better presentation, the trajectory is smoothed with a window size of 10 frames. A significant deformation of the linker, which breaks the hydrophobic core that gating the backdoor channel, can be seen before Pi escaping form the hydrolysis site.

**Movie S3. Pi release from the backdoor channel in eIF4AI with SAT/AAA mutation.** The movie shows a typical Pi release trajectory from the eIF4AI with SAT/AAA mutation. The molecular representation is the same as in movie 1. A deformation of the linker can also be recognized before Pi escaping, although not as strong as in wild type eIF4AI. This is because the backdoor channel is already half-open in the starting structure, which is taken from the cMD simulation results of eIF4AI with SAT/AAA mutation.
